# Irisin combined index to diagnose central precocious puberty in girls: a cross-sectional study

**DOI:** 10.1186/s12887-024-04743-w

**Published:** 2024-04-26

**Authors:** Jie Wang, Yongquan Tang, Guanyu Chen, Mingming Yang, Qian Gao, Yingdan Wang, Wendi Zhou

**Affiliations:** 1 Department of Pediatrics, The Affiliated Huaian No.1 People’s Hospital of Nanjing Medical University, Huaian, 223300 China; 2 Department of Pediatrics, Huai’an Clinical College of Xuzhou Medical University, Huai’an, 223300 China

**Keywords:** Irisin, Pelvic ultrasound, Precocious puberty, Girl, Diagnosis

## Abstract

**Background:**

To investigate serum irisin levels in girls at different developmental status and explore the significance of irisin for the diagnosis of central precocious puberty (CPP) in girls.

**Methods:**

In this cross-sectional study 111 girls were enrolled, including 43 cases of CPP, 44 cases of peripheral precocious puberty (PPP) and 24 cases of girls with normal sexual development as controls. The data on age, weight and height, measured blood levels of luteinizing hormone (LH), follicle stimulating hormone (FSH), estradiol, and irisin were collected. Pelvic Doppler ultrasound was performed to evaluate uterine length, transverse diameter, anteroposterior diameter. The girls were divided into non-CPP group and CPP group according to gonadotropin-releasing hormone (GnRH) stimulation test.

**Results:**

Serum irisin levels were significantly higher in CPP group than in PPP group and normal control group. Serum irisin level was positively correlated with basal LH level, basal FSH level, peak LH level, peak LH /FSH ratio, uterine volume, bone age, and bone age index. The area under the curve, cut-off value, sensitivity and specificity of serum irisin were 0.958, 219.255 pg/ml, 100% and 80.6%. The combined diagnosis of CPP in girls by serum irisin and serum basal LH combined with uterine volume had an AUC, sensitivity, and specificity of 0.994, 97.6%, and 100%, superior to that of the single index.

**Conclusions:**

Serum irisin level in girls with CPP is significantly increased. An irisin combined index could help the diagnosis of CPP in girls.

## Introduction

Precocious puberty refers to the rapid development of internal and external genital organs and the appearance of secondary sexual characteristics before the age of 8 years for girls and 9 years for boys [1]. In the latest precocious puberty diagnosis and treatment guideline in China, the age is defined as 7.5 years old for girls [2]. Based on whether the hypothalamic-pituitary-gonadal axis (HPGA) function is activated, precocious puberty can be classified into central precocious puberty (CPP), peripheral precocious puberty (PPP) and incomplete precocious puberty [1]. The causes of PPP include tumors with abnormal secretion of sex hormones, gonadotropin-secreting tumors, exposure or ingestion of exogenous sex hormones, gene mutations affecting sex hormone production, adrenocortical adenomas, adrenal malignancies, and special syndromes such as Mc-Cune-Albright syndrome. CPP is a common pediatric endocrine disease, which seriously affects the physical and mental health of children. In recent years, the incidence of precocious puberty has been reported to be increasing worldwide, and the age of onset is also higher than before [3–8].

The gonadotropin-releasing hormone (GnRH) provocation test is the gold standard for the diagnosis of CPP [1], but it requires repeated blood sampling, which increases the pain of the children and the economic burden of the parents, and is not easy to be carried out in the outpatient clinic. Therefore, exploring a simple screening method for CPP has become one of the research hotspots in the field of pediatric endocrinology. Irisin is a newly discovered myogenic factor [9]. FNDC5 is the precursor of irisin and is mainly expressed in the muscles, especially in skeletal muscles, while its expression level is low in the kidney, liver and lung [10]. In addition, FNDC5 is mainly expressed in the proximal pituitary. The expression of FNDC5 and irisin in the HPGA axis correlates with developmental and metabolic status and is expressed in a sex-specific manner. Irisin promotes GnRH expression to participate in the regulation of the reproductive system [11, 12]. Irisin was investigated in CPP, but its levels in PPP have not yet been investigated. Therefore, this study aimed to investigate serum irisin in control and girls with CPP and PPP, and to develop an index based on irisin levels for the diagnosis of CPP.

## Methods

### Subjects

This was a cross-sectional study and the sample size was calculated by G-power software. Eighty-seven girls with precocious puberty who attended the pediatric endocrinology clinic of Huai’an First Hospital affiliated with Nanjing Medical University from May 2022 to June 2023 were selected as the study subjects, and 24 girls with normal sexual development during the same period were used as controls. None of the girls were being treated during this study. Inclusion criteria for precocious puberty: girls with breast development before the age of 8 years. Exclusion criteria: (1) precocious thelarche, pubic hair appears early, simple early menarche; (2) previous use of drugs affecting the HPGA axis; (3) poor adherence to the doctor’s instructions. All enrolled children and their parents were fully informed about the study and gave informed consent, and the study was approved by the Ethics Committee of Huai’an First Hospital affiliated with Nanjing Medical University (KY-2023-068-01).

### Data collection

Height, weight, waist circumference (WC), hip circumference, body mass index (BMI) were measured by trained professionals, and height-for-age z-scores (HAZ), weight-for-age z-scores (WAZ) and BMI-for-age z-scores (BAZ) were calculated with the help of the WHO AhthroPlus (version 1.0.4) software [13]. Bone age index (BAI) was calculated as (bone age- actual age) /actual age. Breast staging was based on the Tanner criteria [14]. Serum levels of basal gonadotropin (Gn), estradiol, prolactin (PRL), insulin like growth factor 1 (IGF-1) were measured using electrochemiluminescence immunoassay analyzer with matching kits (Roche). Greulich & Pyle standardized atlas was used to determine bone age. Uterine volume, ovarian volume, number of follicles, and uterine length were assessed by an experienced sonographer with specialized training.

Girls were divided into the CPP group and the PPP group according to the results of GnRH stimulation test: basal values of serum Gn were determined before the stimulation test, and triptorelin acetate (Dabiga, 0.1 mg/branch, Phylin Pharmaceutical Co., Ltd. Germany) was injected subcutaneously with 2.5 µg/kg, maximum 100 µg, and 2 ml of venous blood was withdrawn sequentially at 15, 30, 60, and 90 min after the injection to determine the levels of serum Gn (Immune chemiluminescence method).

### ELISA

The girls fasted for 12 h, and the venous blood was drawn at 8:00 a.m. under a quiet state; the blood samples were centrifuged at 3000 r/min for 10 min. The serum samples were stored at -80℃ in a special refrigerator for specimens. The serum irisin level was determined by ELISA kit (Qiaoyi Company, Anhui, China; cat No: JEN-017).

### Data analysis

SPSS 25.0 software was used for data analysis. Qualitative information was expressed as percentages, and the chi-square test was used to compare two groups. Continuous variables with normal distribution were expressed as mean ± standard deviation (SD), independent sample t-test was used for comparison of two groups, one-way ANOVA was used for comparison of three groups, and Pearson correlation analysis was used for correlation analysis. Non-normally distributed continuous variables were expressed as median (quartile) [M (P_25_, P_75_ )], Man- Whitney U test was used for two-group comparisons, Kruskal-Wallis H test was used for three-group comparisons, and correlation analyses were performed using Spearman rank correlation analysis. Receiver operator characteristic curve (ROC) was plotted, and logistic regression was used to explore the role of single versus combined indicators in the diagnosis of CPP. *P* < 0.05 was considered significant difference, and two-sided tests were used for all tests.

## Results

### General information in three groups of girls

A total of 43 CPP girls, 44 PPP girls and 24 girls with normal sexual development were included. There were significant differences in age, height, WAZ, weight, HAZ, BMI and BAZ among the three groups of girls (*P* < 0.05); there were significant differences in waist circumference, hip circumference, bone age, BAI, IGF-1, basal LH, basal FSH, estradiol, testosterone, uterine volume, ovary volume, peak LH, peak LH /FSH ratio and glucose between CPP and PPP groups (*P* < 0.05), but there were no significant differences in PRL, peak FSH, total cholesterol (TC) and triglycerides (TG) (*P* > 0.05, Table 1). The tanner stages and the percentage of children in each stage were shown in Table 2. CPP and PPP groups had complete data, and normal control group had data on age, height, and weight.

**Table 1:** Comparison of general information of CPP group, PPP group and normal control group

groups	CPP group (*n* = 43)	PPP group (*n* = 44)	Normal control group (*n* = 24)	Z-value	*P*
CA [years, M (P_25_, P_75_ )]	8.42(7.58,8.77)	7.63(6.92,8.17)	7.67(6.33,8.8.25)	-	< 0.001
Height (cm,‾χ ± s)	136.42 ± 9.11	128.73 ± 8.55	122.78 ± 12.17	-	< 0.001
HAZ (‾χ ± s)	1.31 ± 1.03	1.10 ± 1.08	0.32 ± 1.20	-	0.002
Weight [kg, M (P_25_, P_75_ )]	32.85(27.00,41.45)	27.95(24.08,30.00)	24.25(21.05,28.25)	-	< 0.001
WAZ [M (P_25_, P_75_ )]	1.46(0.57,2.09)	1.08(0.44,1.54)	0.09(-0.54,1.31)	-	0.005
BMI (‾χ ± s)	18.15 ± 2.56	17.13 ± 2.20	16.04 ± 2.00	-	0.001
BAZ [M (P_25_, P_75_ )]	1.03(0.14,1.48)	0.78(-0.25,1.29)	0.10(-0.57,0.79)	-	0.029
Waist circumference [cm, M (P_25_, P_75_ )]	65.50(58.00,69.00)	57.60(53.60,63.05)	-	-3.023	0.003
Hip circumference [cm, M (P_25_, P_75_ )]	77.30(68.20,83.00)	69.25(64.28,73.00)	-	-3.757	< 0.001
Bone age [years, M (P_25_, P_75_ )]	11.00(8.83,12.00)	8.83(7.83,8.83)	-	-5.795	< 0.001
Bone age index [M (P_25_, P_75_ )]	0.26(0.19,0.31)	0.13(0.05,0.25)	-	-4.373	< 0.001
IGF-1 [ng/ml, M (P_25_, P_75_ )]	252.80(189.20,408.80)	177.45(147.85,214.23)	-	-4.194	< 0.001
B-LH ≥ 0.3 IU/L [n (%)]	36(83.72)	3(6.82)	-	-	< 0.001
B-FSH [IU/L, M (P_25_, P_75_ )]	3.38(2.37,4.72)	1.76(1.21,2.29)	-	-5.773	< 0.001
Estradiol ≥ 5 Pg/ml [n (%)]	40(93.02)	20(45.45)	-	-	< 0.001
PRL [ng/ml, M (P_25_, P_75_ )]	12.70(7.93,24.30)	11.20(7.82,15.98)	-	-0.785	0.432
Testosterone ≥ 0.087 nmol/l [n (%)]	13(30.23)	3(6.82)	-	-	0.005
P- LH [IU/L, M (P_25_, P_75_ )]	12.25(7.12,19.33)	3.07(2.93,3.67)	-	-3.873	< 0.001
P-FSH [IU/L, M (P_25_, P_75_ )]	12.15(9.72,14.70)	9.77(9.10,11.80)	-	-1.383	0.167
peak LH /FSH ratio [M(P_25_, P_75_)]	1.24(0.54,1.76)	0.31(0.25,0.37)		-3.430	0.001
Uterine volume [cm^3^, M (P_25_, P_75_ )]	2.10(1.90,2.28)	1.10(0.80,1.50)	-	-7.325	< 0.001
Ovarian volume [cm^3^, M (P_25_, P_75_ )]	2.35(1.75,3.45)	2.8(1.60,3.00)	-	-2.566	0.010
Glu[mmol/l, M(P_25_, P_75_)]	4.66(4.56,4.81)	4.95(4.71,5.31)	-	-2.031	0.042
TC[mmol/l, M(P_25_, P_75_)]	3.76(3.30,4.50)	3.76(3.52,4.13)	-	-0.412	0.680
TG[mmol/l, M(P_25_, P_75_)]	0.81(0.60,1.20)	0.78(0.68,1.02)	-	-0.074	0.941

**Table 2:** The percentage of CPP group, PPP group and normal control group in each tanner stage

Tanner stage	CPP group (*n* = 43)	PPP group (*n* = 44)	Normal control group (*n* = 24)
1	0.00%	0.00%	100%
2	55.81%	97.73%	0.00%
3	41.86%	2.27%	0.00%
4	2.33%	0.00%	0.00%
5	0.00%	0.00%	0.00%

Tanner stage 1, when hormones are hard at work behind the scenes; Tanner stage 2, when the first physical signs of puberty occur; Tanner stage 3, the growth spurt stage; Tanner stage 4, the continuation of development; Tanner stage 5, the final stage

### Comparison of serum irisin levels in three groups of girls

**Fig. 1:**
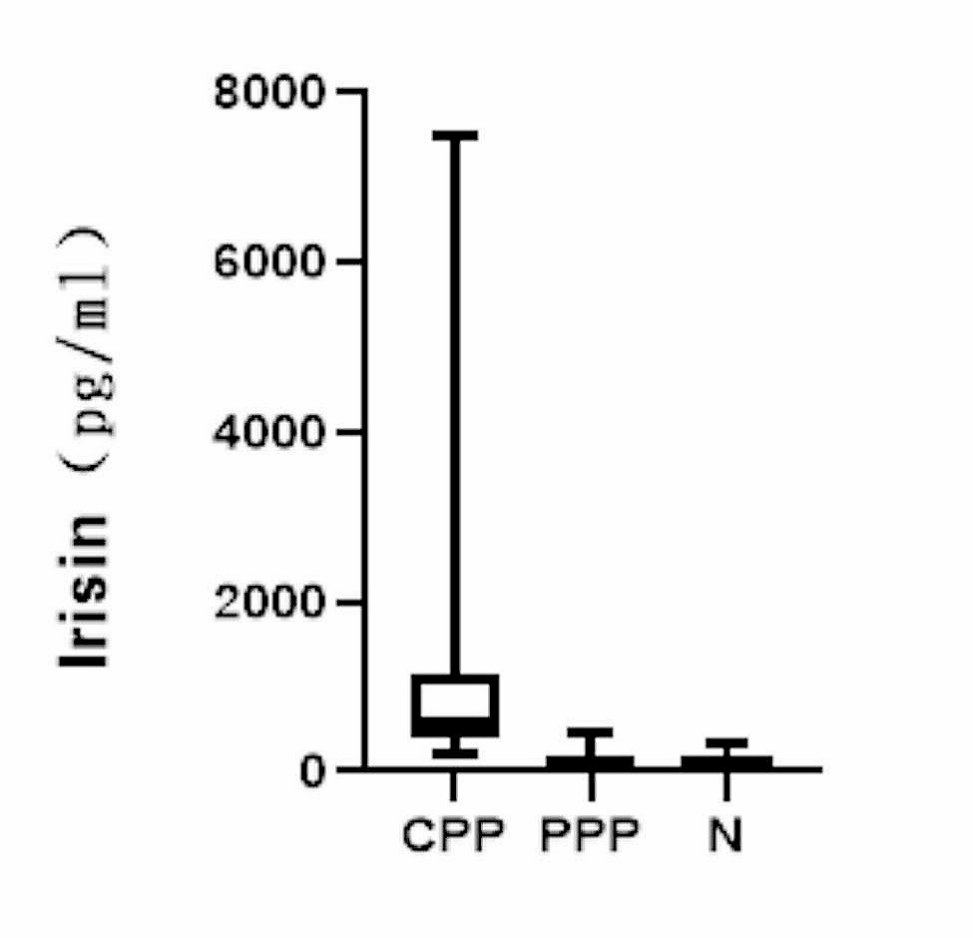
Comparison of irisin levels in CPP group, PPP group and normal control group (N)

The detectable rates of serum irisin in CPP group, PPP group, and normal control group were 95.35%, 70.45%, and 79.17%, and mean irisin level in CPP group 568.61(404.42,1157.14) pg/ml was higher than that in PPP group 118.74(76.81,202.86) pg/ml and normal control group 100.76(52.89,160.75) pg/ml (*P* < 0.05). There was no significant difference in mean irisin level between PPP group and normal control group (*P* > 0.05, Fig. 1).

### Correlation analysis of serum irisin with age, height, weight, blood sex hormones, bone age index and uterine volume in three groups of girls

**Fig. 2:**
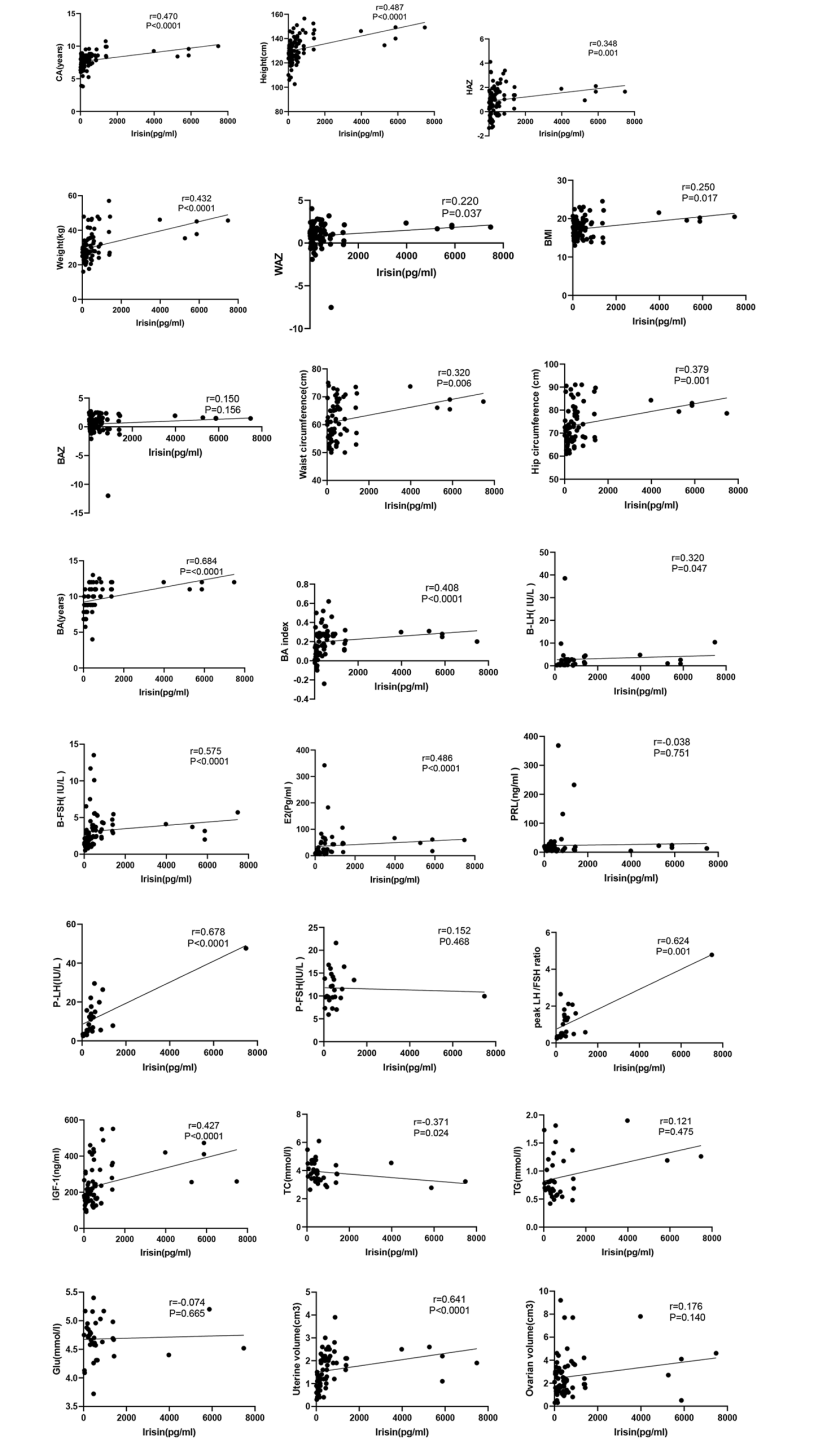
Correlation analysis between irisin and other clinical data

Spearman’s correlation analysis of serum irisin and general clinical data of girls suggested that serum irisin level of girls was positively correlated with age, height, HAZ, weight, WAZ, BMI, waist circumference, hip circumference and bone age, BAI, basal LH and FSH, peak LH, peak LH/FSH ratio, estradiol, IGF-1, uterine volume, but was negatively correlated with total cholesterol (TG) (*P* < 0.05, Fig. 2). No statistical significance was found in the correlation with BAZ, prolactin, peak FSH, ovarian volume, glucose, and triglycerides (*P* > 0.05, Fig. 2). CPP group had 43 girls, PPP group had 44 girls and normal control group had 24 girls. CPP and PPP groups had complete data, and normal control group had data on age, height, weight and irisin.

### Multifactor logistic regression analysis

Whether GnRH stimulation test results were diagnosed as CPP or not was the dependent variable, and multivariate Logistic regression analysis was performed for 43 girls in CPP group and 44 girls in PPP group. There was no significant covariance for the respective variables (VIF < 5). By multifactor regression analysis we established regression model Y = -11.582 + 0.014* irisin + 2.934* serum basal LH + 3.929 * uterine volume (Table 3).

**Table 3:** Logistic regression equation fitting results for serum irisin, serum basal LH and uterine volume

	β	standard error	Wald value	OR value	*P*-value
irisin	0.014	0.007	3.977	1.014	0.046
B-LH	2.934	1.425	4.242	18.806	0.039
uterine volume	3.929	1.660	5.600	50.838	0.018
constant	-11.582	4.289	7.293	0.000	0.007

### ROC curve analysis of serum irisin level and uterine volume in the diagnosis of precocious puberty in girls

ROC curve analysis was performed for 43 girls in CPP group and 44 girls in PPP group. to compare the value of serum irisin level and uterine volume for the diagnosis of CPP in girls. The sensitivity and specificity of basal LH ≥ 0.3 U/L in the diagnosis of CPP were 85.4% and 93.5%, respectively. The cutoff level of irisin at 219.255 pg/ml had a high AUC and sensitivity for the diagnosis of CPP.

**Fig. 3:**
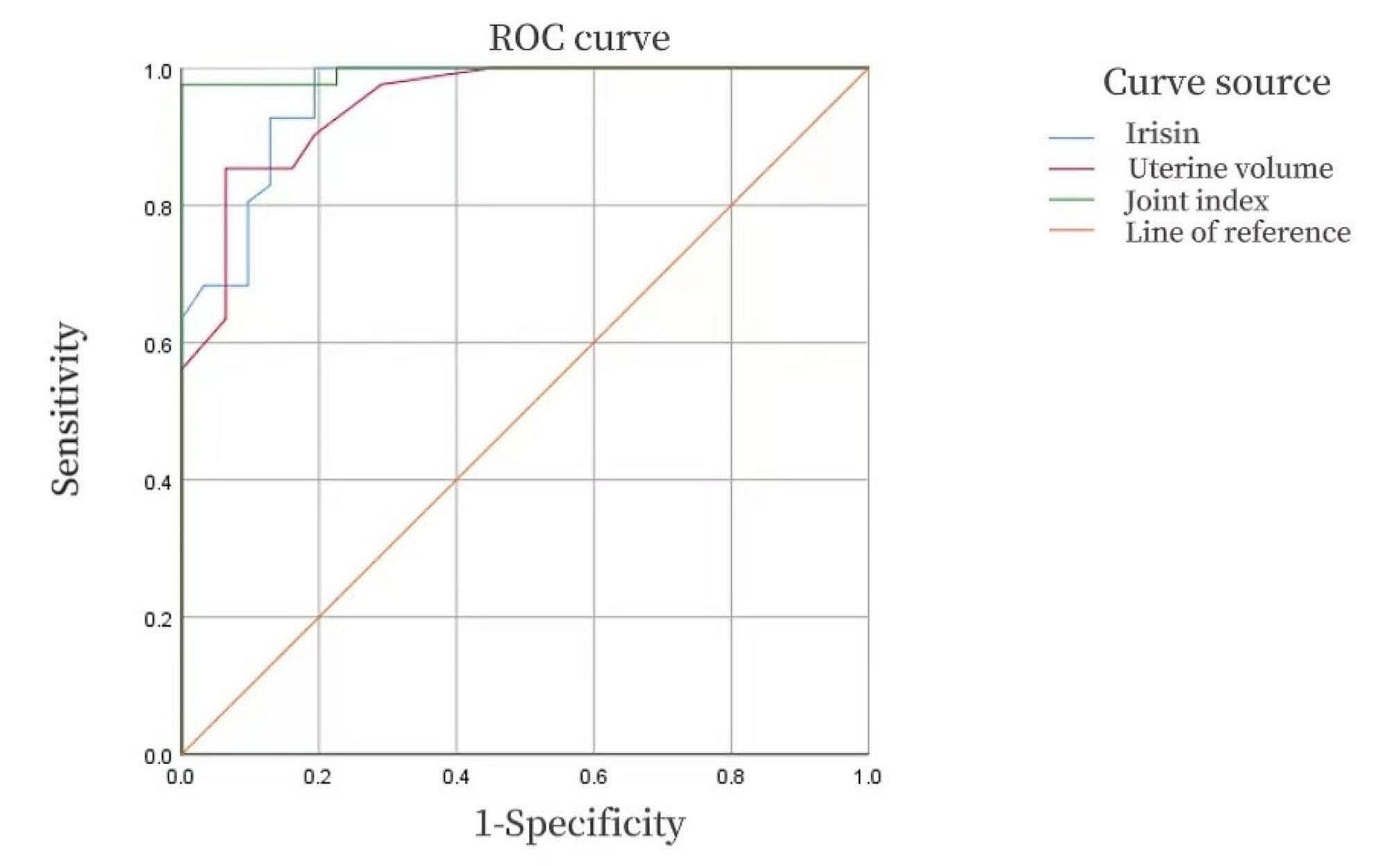
ROC curve of serum irisin, uterine volume and combined indexes for the diagnosis of CPP in girls. ROC curves were based on the analysis of 43 girls in CPP group and 44 girls in PPP group

Serum irisin had a high AUC and sensitivity, and serum LH basal value and uterine volume had a high specificity (Table 4). By multifactor regression analysis we established regression model Y = -11.582 + 0.014* irisin + 2.934* serum basal LH + 3.929 * uterine volume. The above indicators were used as the combined indicators for the diagnosis of CPP, and the ROC curve analysis was performed with the combined index Y. The AUC, sensitivity, and specificity of the model combined with auxiliary diagnosis of CPP were 0.994, 97.6%, and 100%, respectively, which were better than those of single index (Fig. 3). Therefore, the combination of the above three indicators had the highest efficiency in the diagnosis of CPP, and the difference was significant (*P* < 0.05).

**Table 4:** The sensitivity and specificity of serum irisin and uterine volume for the diagnosis of CPP

	AUC	95% CI	bounds	Sensitivity (%)	Specificity (%)
irisin	0.958	0.917–0.999	219.255Pg/ml	100	80.6
uterine volume	0.948	0.902–0.994	1.55 ml	85.4	93.5

## Discussion

Sexual development is controlled by the hypothalamic-pituitary-gonadal axis, which is inhibited in childhood and activated by complex regulatory mechanisms to mark the onset of puberty [15]. Abnormal sexual development such as precocious puberty causes a series of harms to children, such as early menarche in girls, premature epiphyseal closure, which affects the lifelong height, and even psychosocial and behavioral problems such as anxiety and depression [16, 17]. Recent studies have shown that early menarche increases the incidence of postmenopausal breast cancer and the risk of type 2 diabetes and cardiovascular disease [18–20]. Epidemiological studies have shown that early puberty in women or men is associated with a higher incidence of diseases of the reproductive system, endocrine system, circulatory system, digestive system and nervous system [21, 22]. Therefore, early diagnosis and treatment of precocious puberty have important significance.

Currently, basal LH levels are usually used in clinical practice to assess HPGA activity. The European guideline consensus update suggests that HPGA initiation can be determined when the basal luteinzing hormone (LH) value is greater than 0.83 [23]. However, GnRH provocation test is still required to clarify the type of precocious puberty in most children [24]. However, the results of GnRH stimulation test are affected by obesity and other factors, and false-negative phenomenon may occur in the clinic. Therefore, there is an urgent need to develop diagnosis methods of precocious puberty that are not affected by obesity and other factors.

It has been shown that irisin levels are associated with puberty, and irisin levels are significantly correlated with Tanner staging [25, 26]. The irisin precursor, type III fibronectin component-containing protein 5 (FNDC5), is mainly expressed in the proximal pituitary gland, and is upregulated during puberty to promote the expression of LH and follicle-stimulating hormone (FSH) [27]. In present study, serum irisin levels were significantly higher in girls with CPP than in girls with PPP and normal sexual development, suggesting that irisin level may be useful for the initial screening of precocious puberty.

A growing number of studies have shown that obese children are prone to precocious puberty, and basal LH levels and peak LH excitation in obese girls are significantly lower than in girls with normal BMI [28, 29]. BMI is correlated with body fat and is not affected by height. In present study, we found that serum irisin levels did not correlate with BAZ in girls with CPP. Therefore, irisin may exclude the interference of obesity in the diagnosis of CPP. Uterine volume and serum Gn levels are known to be helpful in the diagnosis of precocious puberty, but the reliability is not good and the interference of factors such as obesity cannot be excluded. In this study, serum irisin, serum basal LH and uterine volume were combined to diagnose CPP in girls, which were better than a single index.

This study showed a high correlation between irisin and bone age and BAI. Irisin can be higher secondarily to the higher bone age of CPP girls, as Irisin and bone age have the higher correlation among the variables studied. Recent evidences suggest that irisin is involved in the regulation of bone state [30]. Bone age examination is known to be radioactive and cannot be repeated in a short period of time. We will further study the relationship between irisin and bone age in CPP and PPP groups in order to reduce the exposure of radioactive substances in the treatment of children with precocity.

## Conclusion

Although this study has limitations as a single-center study with a small number of cases, and we did not monitor the changes of irisin levels during the follow-up of CPP treatment, our study was the first report on the use of irisin combined index to diagnose CPP. Our results demonstrate that serum irisin level has no correlation with BAZ value, and can improve the diagnosis of CPP when it is combined with serum basal LH and uterine volume.

## Data Availability

All data are available upon request to correspondence authors.
